# Clinical Impact of Rare Subtypes of Parathyroid Adenoma: A Systematic Review

**DOI:** 10.3390/jpm16040211

**Published:** 2026-04-10

**Authors:** Maurizio Martiradonna, Rossella Mazzilli, Virginia Zamponi, Daniela Prosperi, Massimiliano Mancini, Antongiulio Faggiano

**Affiliations:** 1 Endocrinology Unit, Department of Clinical and Molecular Medicine, Sant’Andrea Hospital, ENETS Center of Excellence, Sapienza University of Rome, 00189 Rome, Italy; maurizio.martiradonna@uniroma1.it (M.M.); rossella.mazzilli@uniroma1.it (R.M.); virginia.zamponi@uniroma1.it (V.Z.); 2 Nuclear Medicine Unit, Sant’ Andrea University Hospital, 00189 Rome, Italy; dprosperi@ospedalesantandrea.it; 3 Department of Histopathology, Sant’ Andrea University Hospital, 00189 Rome, Italy; mamancini@ospedalesantandrea.it

**Keywords:** hyperparathyroidism, parathyroid adenoma, parathyroid lipoadenoma, parathyroid oncocytic adenoma, oxyphilic adenoma, water-clear cell parathyroid adenoma (WCCA), calcium, parathyroid hormone (PTH)

## Abstract

**Background:** Parathyroid lipoadenoma, oncocytic parathyroid adenoma, and water-clear cell parathyroid adenoma (WCCA) are exceptionally rare subtypes of parathyroid adenoma, whose real clinical impact remains unclear. **Methods**: We performed a literature review and comparison of these uncommon subtypes of parathyroid adenoma, as these lesions may be associated with distinct clinical, pathological and radiological phenotypes. **Results**: The three groups were comparable in terms of age and gender, mainly affecting middle-aged women. As for the biochemical findings, although PTH level did not show statistically significant differences among the three adenomas, in the two-tail comparison between WCCAs and lipoadenomas, there was a trend towards higher PTH values (*p* = 0.058). However, serum calcium levels differed significantly, with higher levels in WCCAs than in lipoadenomas (12.1 vs. 11.3 mg/dL; *p* = 0.002). From a preoperative point of view, ultrasound positivity was significantly higher in WCCAs than in lipoadenomas and oncocytic adenomas (97% vs. 50% vs. 55.3%, *p* < 0.001), compared to scintigraphy positivity, which was comparable between subtypes and relatively suboptimal (66.7% vs. 64.3% vs. 61.3%; *p* = 0.825); WCCAs also had an overall more voluminous morphological phenotype. **Conclusions**: This review, although limited by its reliance primarily on case reports and small case series, confirms that lipoadenoma, oxyphilic adenoma, and WCCAs are rare but clinically relevant subtypes of parathyroid adenoma. These entities may be associated with atypical presentations of primary hyperparathyroidism (including difficult preoperative localization and clinicopathological features raising suspicion of malignancy) and should be recognized as a potential source of diagnostic difficulty. A better understanding of these rare subtypes may improve clinicopathological interpretation, increase awareness of potential diagnostic pitfalls and support a more personalized diagnostic and surgical approach in the future.

## 1. Introduction

Primary hyperparathyroidism (PHPT) is an endocrine disorder characterized by the excessive secretion of parathyroid hormone (PTH) by one or more parathyroid glands, resulting in elevated serum calcium levels. Most cases are attributed to common etiologies such as parathyroid chief-cell adenomas, or, less commonly, parathyroid carcinoma [1]. Most of these cases are sporadic, with a smaller percentage being genetic or related to endocrine syndromes [2]. Surgery is the definitive and curative treatment [3]; however, in select patients, observation alone or medical therapy could be considered appropriate [4,5]. However, there are rare causes, including lipoadenoma, oxyphilic adenoma, and water-clear cell adenoma (WCCA), that may complicate the diagnostic work-up and subsequent management. As reported in the “Overview of the 2022 WHO Classification of Parathyroid Tumors” [6], parathyroid lipoadenoma is defined as a neoplasm characterized by parathyroid parenchymal proliferation and an abundant component of mature adipocytes, with >50% of the glandular volume consisting of adipose tissue and is often associated with abnormal glandular weight.

Furthermore, oxyphilic or oncocytic parathyroid adenoma is defined as a lesion in which >75% of neoplastic cells have oncocytic (or oxyphilic) features, with abundant granular eosinophilic cytoplasm, and is described as large adenomas with possibly higher preoperative calcium levels and higher likelihood of localization with Sestamibi scan [6].

Finally, WCCAs are described as composed entirely of “water clear” cells, in which the cytoplasm is vacuolated, most likely because of glycogen accumulation, with hyperchromatic nuclei [6].

Due to the rarity of these entities, the heterogeneity of diagnostic criteria across studies, and the predominance of case reports or small case series, their real clinical impact remains unclear. Accordingly, we performed a literature review and comparison of these uncommon subtypes of parathyroid adenoma, as these lesions may be associated with distinct clinical, pathological and radiological phenotypes, and a clearer definition of their clinicopathological profile may support a more personalized approach to diagnosis and surgical management.

## 2. Materials and Methods

This systematic review was conducted in accordance with the PRISMA 2020 statement. The review protocol was not prospectively registered in PROSPERO or in any other international database. A literature search was conducted on PubMed/MEDLINE independently by two reviewers. Exclusion criteria were: (a) published in languages other than English; (b) duplicate publication; (c) no data available behind the title and abstract to be evaluated.

For the lipoadenoma and oncocytic parathyroid subtype, the keywords “parathyroid lipoadenoma”, “oncocytic parathyroid adenoma”, or “oxyphyl parathyroid adenoma” were used to identify case reports and case series published between January 1985 and January 2026.

**Figure 1:**
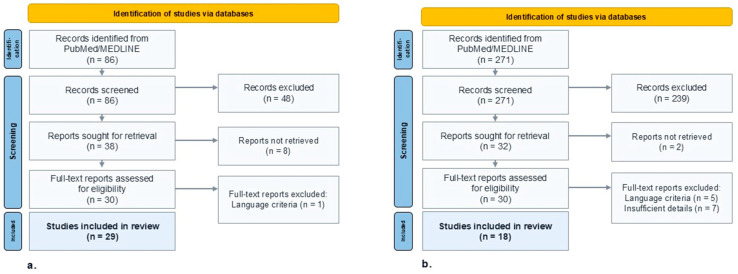
(**a**) Flowchart for parathyroid lipoadenoma; (**b**) flowchart for oncocytic parathyroid adenoma.

For the former, the search returned 86 records. After screening, 1 article was excluded due to language criteria, 8 articles were excluded due to unavailability of the full text, and 48 were excluded due to irrelevance. A total of 29 studies were included (Figure 1a). In addition, cases were included when reported as parathyroid lipoadenoma based on histological diagnosis; when available, confirmation of the cut-off ≥50% of adipose component [6] was searched for, and cases with an explicitly <50% percentage were excluded. In studies where the percentage of adipose tissue was not specified, the case was included if the authors clearly attributed the diagnosis of lipoadenoma, assuming adherence to the most common classifications. For greater data homogeneity, parathyroid lipohyperplasia and other variants/entities that were not fully comparable with lipoadenoma were excluded.

For the latter, the search yielded 271 records. After screening and evaluation of the full text, 5 articles were excluded due to language criteria, 2 due to unavailability of the full text and 246 were excluded as out of scope, of which 239 were irrelevant, and 7, although dealing with oncocytic lesions, reported insufficient clinical, biochemical or histological details for data extraction. A total of 18 studies were therefore included (Figure 1b). Cases were considered oncocytic or oxyphilic when the histology described a lesion predominantly consisting of oxyphilic or oncocytic cells; where possible, the commonly used cut-off of >75% oncocytic cells in the tumor was applied [6], excluding cases for which a lower cut-off was specified. Finally, forms associated with tertiary hyperparathyroidism and parathyroid carcinomas were also excluded.

Lastly, for the WCCA subtype, given the presence of a review with research up to 2021 (January 1985–August 2021) [7], an update approach was adopted. Specifically, a search was performed on PubMed/MEDLINE up to January 2026 to identify new case reports or case series published after the period covered by the previous review. Through the update, five more recent cases were identified and included. To these, we added data from our institutional case, which was described separately as a present case and therefore does not come from bibliographic research. In a number of case series, clinical and biochemical data (sex, age, calcium levels, PTH, lesion size or weight) were not reported for individual patients but only in aggregate form (mean/median). These studies were still included in the review to save relevant information and to represent the overall number of cases. Nevertheless, to avoid mixing units of analysis, quantitative summaries were mainly performed on patient-level data.

For each analyzed variable, *n* refers to the number of cases for which data were available. Categorical variables were reported as percentages and compared between groups using the chi-square (χ^2^) test. Given the nature of the data, the asymmetry of the distributions and the presence of outliers, continuous variables were summarized as median and interquartile range (Q1–Q3) and compared between two independent groups using the Mann–Whitney U test. The Kruskal–Wallis test was used to compare ages between the three subtypes. A *p*-value < 0.05 was considered statistically significant (95% confidence level). Subgroup analyses, sensitivity analyses, and formal meta-analytic procedures, as well as formal risk-of-bias assessment, reporting-bias assessment, and certainty-of-evidence evaluation, were not feasible due to the small number of cases and the heterogeneity of the reports included. The statistical analysis was performed by IBM SPSS (Version 27.0, SPSS Inc. Chicago, IL, USA).

## 3. Results

The **lipoadenoma subgroup** included 29 studies involving a total of 55 patients. There was a clear female predominance (60%), and the median age was 58.5 years (IQR 50–66; *n* = 50) (Table 1). Biochemical values showed a median PTH of 146 pg/mL (IQR 97.35–312.50; *n* = 36) and a median calcemia of 11.3 mg/dL (IQR 10.8–12.01; *n* = 48). The reported tumor dimensions had a median of 22.5 mm (IQR 15–51; *n* = 30) and the weight a median of 659.5 mg (IQR 322–3000; *n* = 30) (Table 2). From a diagnostic point of view, ultrasound was positive in 20/40 cases (50%), while Sestamibi scintigraphy was positive in 26/39 (66.7%) and CT in 16/19 (84.2%) (Table 3). Ectopic localization was described in 5/55 cases (9.09%). Body mass index (BMI), available in a limited number of patients, had a median of 27.3 kg/m^2^ (IQR 21.8–34.55; *n* = 9). The **oncocytic/oxyphilic subgroup** included 18 studies, for a total of 142 patients, with a female prevalence of 68% (Table 1). However, the availability of patient-level data was scarce: age was reported for 33 patients with a median of 58 years (IQR 44–63), calcemia for 31 patients (median 11.8 mg/dL, IQR 11–12.7), and PTH for 15 (median 211.4 pg/mL, IQR 112–827). The dimensional characteristics showed a median size of 25 mm (IQR 16–31; *n* = 27) and a median weight of 2090 mg (IQR 944.5–3240; *n* = 16) (Table 2). In terms of diagnosis, ultrasound was positive in 55.3% (68/123) and scintigraphy in 61.3% (73/119), while CT was positive in 83.3% (5/6) (Table 3). Lastly, the **water-clear cell adenoma subgroup** included 34 studies, for a total of 43 patients, with a female prevalence of 67.4%. The median age was 56 years (IQR 48–70; *n* = 43) (Table 1). From a biochemical point of view, the median calcemia was 12.1 mg/dL (IQR 11.6–12.6; *n* = 33) and the median PTH was 303 pg/mL (IQR 130–489; *n* = 39). The median lesion size was 37.5 mm (IQR 28–50; *n* = 32), and the median weight was 5340 mg (IQR 900–13,300; *n* = 31) (Table 2). Regarding imaging, ultrasound was positive in 32/33 cases (97%), while Sestamibi scintigraphy was positive in 18/28 (64.3%) (Table 3). In some cases, particularly in older reports, preoperative imaging was inconclusive, and the adenoma was eventually detected during surgical neck exploration.

By comparing the three subtypes, female gender and age were comparable across the three groups, with no significant differences (women: 60.0% vs. 67.4% vs. 67.6%; *p* = 0.583; median age: 58.5 vs. 56 vs. 58 years; *p* = 0.358, Table 1).

PTH values did not show statistically significant differences in two-tailed comparisons (lipoadenoma vs. water-clear *p* = 0.058; lipoadenoma vs. oncocytic *p* = 0.260; water-clear vs. oncocytic *p* = 0.931, Table 2) as well. In contrast, calcemia was higher in the water-clear group than in the lipoadenoma group (12.1 [11.6–12.6] vs. 11.3 [10.8–12.01] mg/dL; *p* = 0.002, Table 2), while no significant differences were found between lipoadenoma and oncocytic (*p* = 0.065) or between water-clear and oncocytic (*p* = 0.375) groups.

In terms of size, no differences were observed between lipoadenoma and water-clear (*p* = 0.131) or between lipoadenoma and oncocytic (*p* = 0.737) groups, while the water-clear subtype presented significantly larger lesions than the oncocytic subtype (37.5 [28–50] vs. 25 [16–31]; *p* = 0.001, Table 2). Finally, weight was larger in the water-clear group than in the lipoadenoma group (5340 [900–13,300] vs. 659.5 [322–3000]; *p* = 0.005) and compared to the oncocytic group (*p* = 0.034), with no differences between lipoadenoma and oncocytic groups (*p* = 0.189) (Table 2).

In Table 3, ultrasound positivity differs significantly between subtypes: it is very high in the water-clear cells group (32/33; 97.0%) compared to the lipoadenoma (20/40; 50.0%) and oncocytic groups (68/123; 55.3%), with a statistically significant difference (χ^2^, *p* < 0.001). Conversely, positivity on scintigraphy techniques is comparable between groups (66.7% vs. 64.3% vs. 61.3%; *p* = 0.825, Table 3). CT was positive with high percentages in the groups with available data (lipoadenoma 84.2%; oncocytic 83.3%), but the comparison between subtypes was not performed due to the absence of data in the water-clear cells group.

### Case Report

Alongside this review, we report an institutional case of water-clear cell parathyroid adenoma. A 30-year-old female patient was admitted to the Endocrinology Unit of the Sant’Andrea Hospital due to the accidental detection of high PTH and alkaline phosphatase (ALP) levels in routine tests, requested by her general practitioner. The patient had a family history of thyroid disease and cancer (father with testicular lymphoma), no personal history of major diseases, no history of nephrolithiasis and appeared to be asymptomatic; BMI and blood pressure were 19 kg/m^2^ (normal weight) and 115/70 mmHg, respectively. Blood and urine tests were repeated, and the laboratory data showed serum calcium, 12.08 mg/dL (normal range 8.48–10.06 mmol/L); serum PTH, 1084.7 pg/mL (normal range 15–68.3 pg/mL); ALP, 182 UI/L (45–115 UI/L); and 25-OH vitamin D, 8.3 ng/mL (normal range > 30 ng/mL). The biochemical picture with increased serum concentrations of PTH, even after correction of 25-OH vitamin D levels, hypercalcemia, increased ALP and hypophosphatemia, was consistent with the diagnosis of primary hyperparathyroidism. Neck ultrasound showed a large hypoechoic non-homogeneous area, 2.8 cm in size, with a central anechoic area, located at the inferior part of the left thyroid lobe. Perilesional vascularization was also highlighted. 18F-choline PET-CT showed increased uptake of the lesion located at the inferior-posterior side of the left thyroid lobe with SUVmax 5.41. To better define the lesion, a Magnetic Resonance Imaging (MRI) of the neck with contrast enhancement was performed, describing an area of 3.2 × 3.0 cm below the left lobe of the thyroid with a mixed solid-fluid appearance. A dual-energy X-ray absorptiometry (DEXA) scan revealed lumbar osteopenia and femoral osteoporosis. The markedly high serum concentration of PTH, the size of the lesion and its PET uptake were reasonably in favor of a suspected parathyroid carcinoma rather than adenoma, and the decision was to extend surgery. The patient underwent surgical removal of the lesion and left hemithyroidectomy. Surprisingly, the histological report was WCCA of the lower left parathyroid, as the parathyroid gland was completely replaced by a monomorphic proliferation of medium-sized cells with clear cytoplasm, forming a dense population with minimal intervening loose connective tissue, and at high magnification, the tumor cells exhibited no significant nuclear atypia, mitotic figures, or other features suggestive of malignancy.

## 4. Discussion

Parathyroid lipoadenoma, oncocytic parathyroid adenoma, and water-clear cell parathyroid adenoma are exceptionally rare subtypes of parathyroid adenoma. Therefore, no reliable prevalence estimates are available, but wide histopathological series report institutional prevalences of approximately 0.20% for lipoadenoma [8] and 0.15% for WCCAs [9], while for oncocytic adenoma, the frequency varies widely depending on histological criteria and in a large surgical cohort was around 3.3% [10]. Regarding parathyroid lipoadenoma, the results of the present review highlight the size of these tumors, as described in the Overview of the 2022 WHO Classification of Parathyroid Tumors [6], with a median size of 22.5 mm and median weight of 659.5 mg, as well as reports of giant forms. From a biochemical point of view, lipoadenomas typically present with a picture compatible with hypercalcemic hyperparathyroidism; the results of this review confirm this pattern, with a median calcemia of 11.3 mg/dL and a median PTH of 146 pg/mL. Furthermore, in a limited number of patients for whom data were available, BMI showed a median of 27.3 kg/m^2^ (IQR 21.8–34.55; *n* = 9), suggesting, with all caution associated with small sample size, a possible higher representation of overweight subjects. The most significant aspect of this subtype of parathyroid adenoma is undoubtedly its preoperative localization, which is a crucial step in surgical planning, particularly when considering minimally invasive approaches. In lipoadenomas, the rich adipose component can be a potential obstacle to this. In this regard, Fujimoto’s study [11] explored the impact of fat content on ultrasound performance, observing a reduction in sensitivity as the fat content increased, with improvement when additional imaging methods were integrated. The authors also described a possible ultrasound phenotype of lipoadenoma, which, compared to the “classic” adenoma, may present less distinct margins, iso- or hyperechoic echogenicity, heterogeneity with a “two-tone pattern,” reduced vascularization, and, more frequently, absence of the polar artery, as well as less clearly mappable vascularization. The data extracted in this review are consistent with suboptimal ultrasound performance in lipoadenoma: ultrasound was positive in 50% of cases (20/40). Sestamibi scintigraphy also showed only moderate performance, with positivity in 66.7% (26/39), suggesting that the method may not be sufficiently reliable as the sole localization test in this specific variant. In contrast, neck CT would seem to offer a more consistent contribution (84.2% positive, 16/19), potentially also in relation to the size of these tumors. Currently, data on the role of PET methods such as PET/CT with 18F-choline, which is increasingly used at present [12], is almost absent, making its potential diagnostic value in this population indeterminable. A further element to consider is ectopic localization, documented in approximately 9.1% of cases (5/55), frequently mediastinal, which may contribute to the difficulty of preoperative identification, especially when first-level imaging is negative or inconclusive. However, reporting bias must be considered, as it could have overestimated this percentage. Regarding oncocytic parathyroid adenoma, the results of this review are also consistent with the “Overview of the 2022 WHO Classification of Parathyroid Tumors” [6], describing a tendency toward large lesions (median size of 25 mm and median weight of 2090 mg) and significant hypercalcemic hyperparathyroidism, despite having been considered non-functioning adenomas for a long time until the late 1970s [13]. In support of this anatomopathological and biochemical presentation, a clinically relevant element that emerged from the cases was the occurrence of preoperative suspicion of parathyroid carcinoma in at least five reports, indicating that, in some patients, the clinical and imaging presentation of oxyphil adenomas may overlap with features raising concern for malignancy. It is widely recognized that distinguishing between parathyroid adenoma and carcinoma is difficult due to similar clinical characteristics, but accurate prediction of malignancy is essential to guide appropriate therapeutic decisions, particularly from a surgical point of view. In fact, the preferred treatment is *en bloc* surgical resection, while management of recurrent disease is based on reoperation, when practicable, and on medical control of hypercalcemia with calcimimetics and antiresorptive agents, since chemotherapy and radiotherapy have shown limited efficacy, whereas newer targeted approaches such as tyrosine kinase inhibitors, temozolamide and immune checkpoint inhibitors have shown promising but limited evidence of efficacy [14,15]. Although the identification of metastases is the main indicator of cancer prior to histological analysis, certain factors may lead to suspicion of one or the other, such as albumin-corrected calcium > 3 mmol/L, PTH > 3 times the upper limit of the normal range and, finally, a parathyroid lesion > 3 cm on ultrasound or a palpable mass in the neck (>3 cm) in hypercalcemic patients with primary hyperparathyroidism or with concomitant severe renal and skeletal involvement [16]. However, our dataset did not allow for robust conclusions about renal or skeletal complications, due to the intrinsic heterogeneity of case reports, as well as the absence of standardized information such as imaging for nephrolithiasis or bone densitometry (BMD) measured by MOC DXA. With regard to osteoporosis, in a recent study, Zhang and colleagues [17], applied single-cell RNA sequencing to resected parathyroid tissue in patients with PHPT with and without osteoporosis, showing a greater abundance of oxyphilic cell subpopulations and a more intense cellular communication network in subjects with osteoporosis, with specific oxyphilic subclasses (e.g., HSPA1A-OC/SPARCL1-OC) more involved in interactions, as well as describing the upregulation of signaling pathways potentially relevant to the bone phenotype including WNT/β-catenin. Although it was not a study dedicated to oncocytic adenoma, these data shed light on innovative aspects of this peculiar cell type and may, in the future, provide biological support for translational studies to explore a possible link between oxyphilic predominance and bone fragility. The molecular aspect of oncocytic adenoma was also studied by Lu and colleagues [18], who analyzed a cohort of 664 sporadic parathyroid adenomas, showing that in terms of the gene expression of CASR, VDR, FGFR1, CDKN1B, MEN1, and others, there were no significant differences between classic adenomas and oxyphilic adenomas, while VDR (vitamin D receptor) protein expression was weaker in oxyphilic adenomas. Proteomic differences involved in functional regulation mechanisms and tumorigenesis through p53 also emerged. From an imaging perspective, in our review, the positivity of scintigraphy with Sestamibi was 61.3%, lower than expected for a lesion rich in mitochondria. Physiologically, Sestamibi is known to concentrate mainly in mitochondria [19], which are abundant in oxyphilic cells [20]. However, clinical evidence is controversial. Earlier studies suggested a higher probability of delayed uptake in the presence of a high oxyphilic fraction of the parathyroid adenoma [21]; however, subsequent studies, such as that by Kobylecka [22], indicate that Sestamibi uptake may correlate with gland size and overall functional status, but not with the percentage of oxyphilic cells alone. Similar to other rare variants, data on the role of PET methods, particularly PET/CT with 18F-choline, are scarce or anecdotal for oncocytic adenoma, making their potential diagnostic value in this population currently undefined. Finally, a particular issue concerns the differential diagnosis in cases of intrathyroid parathyroid (five cases in our dataset), where differentiation from thyroid oncocytic neoplasms may be challenging. In this scenario, fine needle aspiration showing oxyphilic cells is not inherently discriminatory. Some authors have proposed useful cytomorphological criteria, suggesting that oncocytic thyroid neoplasms have much larger nuclei, more often prominent nucleoli, and the cells tend to be more dyscohesive. In clinical practice, immunocytochemistry (PTH vs. thyroglobulin) and PTH measurement in needle washout fluid could also be highly informative to confirm parathyroid origin [23,24].

Finally, WCCAs are often larger than chief cell adenomas [6] and are thought to have low endocrinological activity; therefore, it is not until the adenoma reaches a significant size and weight that biochemical markers display highly abnormal values [25]. In this review, WCCAs were indeed large (median size 37.5 mm), heavy (median weight 5340 mg), and were associated with particularly marked PHPT, with a median serum calcium of 12.1 mg/dL and a median PTH of 303 pg/mL. These findings, which are consistent with our institutional case, may suggest that, like oncocytic adenoma, WCCAs might also present with features that mimic parathyroid carcinoma. Hence, our case seems to fit well within the clinicopathological spectrum emerging from this review. From an imaging standpoint, WCCAs showed great detectability on ultrasound, which was positive in 97% of cases (32/33). In contrast, Sestamibi scintigraphy demonstrated moderate sensitivity, with positivity in 67% (18/28), comparable to that we observed for lipoadenoma and oxyphil adenomas. Lastly, an additional distinctive feature was the occurrence of double lesions (and even triple lesions [26]), as it was reported in 5/43 patients (11.6%), not commonly encountered in the other two subtypes.

As already mentioned in the results section, when comparing subtypes, the groups were comparable in terms of age and gender, mainly affecting middle-aged women (Table 1). Although PTH did not show statistically significant differences, in the comparison between WCCA and lipoadenoma, there was a trend towards higher PTH values (*p* = 0.058). Calcemia differed significantly, with higher values in WCCAs than in lipoadenomas (Table 2). From a preoperative point of view, ultrasound positivity was significantly higher in WCCAs, compared to scintigraphy positivity, which was similar between subtypes; for CT, the comparison is limited by incomplete data (Table 3). Lastly, WCCAs had a more voluminous morphological phenotype (larger size and weight) (Table 2). Future studies based on larger cohorts with a more comprehensive reporting of clinically relevant complications as well as molecular profiling, may help support a more tailored approach to patient management, also clarifying their impact on patient-specific outcomes, including renal, skeletal and cardiovascular manifestations.

## 5. Study Limitations

This review has several limitations. The available evidence is based mostly on case reports and case series, which limits the generalizability of these findings. Therefore, these results should be interpreted with caution due to the numerical heterogeneity across groups, missing biochemical, radiological or pathological data in some reports and possible publication bias and bias arising from multiple comparisons. Also, it is reasonable that improvements in imaging technology and operator expertise may have improved detection over time, but this could not be effectively assessed in this review.

## 6. Conclusions

This review confirms that lipoadenoma, oxyphilic adenoma, and WCCAs are rare but clinically relevant variants of parathyroid adenoma, associated with hypercalcemic PHPT and whose diagnostic profile could differ between subtypes and may influence the localization process. In lipoadenoma, the biochemistry profile is comparable to “classic” PHPT, but preoperative localization seems to be more complex, likely due to the high adipose component. Oxyphilic adenoma tends to present with large, heavy lesions with elements that might raise preoperative suspicion of carcinoma, highlighting the need for accurate clinical and radiological assessment. The sensitivity of Sestamibi scintigraphy, although theoretically favored by the high mitochondrial density of oxyphilic cells, appears to be suboptimal. WCCAs can also be associated with a marked biochemical and morphological phenotype, which can generate preoperative suspicion of carcinoma, and with excellent ultrasound detectability (≈97% in cases with available data) but only moderate scintigraphic sensibility. Considering the rarity of these subtypes of parathyroid adenoma, our knowledge would benefit from larger, multicenter case series in order to study their clinical profile more robustly (particularly renal failure, osteoporosis, and nephrolithiasis), adopting systematic and standardized data collection, including renal function (eGFR), BMD, and a structured assessment of nephrolithiasis (ultrasound or abdominal CT). Furthermore, it would be desirable to investigate the role of PET/CT with 18F-choline in preoperative localization, which to date has been scarcely investigated in these subtypes. In conclusion, the more we learn about these rare entities, the more their recognition may lead to a more tailored and patient-specific approach to their diagnostic and therapeutic management.

## Figures and Tables

**Table 1:** Comparison of population characteristics in lipoadenoma, water clear and oncocytic parathyroid adenoma.

	Lipoadenoma	Water-Clear	Oncocytic	*p*
Number of patients	55	43	142	
Females (%)	60	67.4	67.6	0.583 *
Median age (Q1–Q3)	58.5 (50–66) (*n* = 50)	56 (48–70) (*n* = 43)	58 (44–63) (*n* = 33)	0.358 **

* Chi-square test. ** Kruskal-Wallis’ test.

**Table 2:** Comparison of main characteristics between lipoadenoma, water-clear cells and oncocytic parathyroid adenoma.

	Median PTH pg/mL (Q1–Q3)	Median Serum Calcium mg/dL (Q1–Q3)	Median Size mm (Q1–Q3)	Median Weight mg (Q1–Q3)
Lipoadenoma	146 (97.35–312.5) *n* = 36	11.3 (10.8–12.01) *n* = 48	22.5 (15–51) *n* = 30	659.5 (322–3000) *n* = 30
Water-Clear	303 (130–489) *n* = 39	12.1 (11.6–12.6) *n* = 33	37.5 (28–50) *n* = 32	5340 (900–13,300) *n* = 31
Oncocytic	211.4 (112–827) *n* = 15	11.8 (11.0–12.7) *n* = 31	25 (16–31) *n* = 27	2090 (944.5–3240) *n* = 16
Lipoadenoma vs. Water-Clear (*p*)	0.058 *	**0.002** *	0.131 *	**0.005** *
Lipoadenoma vs. Oncocytic (*p*)	0.260 *	0.065 *	0.737 *	0.189 *
Water-Clear vs. Oncocytic (*p*)	0.931 *	0.375 *	**0.001** *	**0.034** *

* Mann-Whitney test. Significant *p*-values shown in bold.

**Table 3:** Imaging comparison between lipoadenoma, water-clear cells and oncocitic parathyroid adenoma.

	Positive on Ultrasound	Positive on Scintigraphy	Positive on CT
Lipoadenoma	20/40 (50.0%)	26/39 (66.7%)	16/19 (84.2%)
Water-Clear Cells	32/33 (97.0%)	18/28 (64.3%)	n.a.
Oncocytic	68/123 (55.3%)	73/119 (61.3%)	5/6 (83.3%)
*p*	<0.001 *	0.825 *	—

* Chi-square test. Significant *p*-values are shown in bold. n.a. = not available.

## Data Availability

No new data were created or analyzed in this study. Data sharing is not applicable to this article.
